# The circular RNA circEIF3M promotes breast cancer progression by promoting cyclin D1 expression

**DOI:** 10.18632/aging.103539

**Published:** 2020-07-11

**Authors:** Xiujuan Li, Zhaojun Ren, Yufeng Yao, Jun Bao, Qiao Yu

**Affiliations:** 1 Department of General Surgery, Jiangsu Cancer Hospital, Jiangsu Institute of Cancer Research, The Affiliated Cancer Hospital of Nanjing Medical University, Nanjing, China; 2 Department of Pathology, Jiangsu Cancer Hospital, Jiangsu Institute of Cancer Research, The Affiliated Cancer Hospital of Nanjing Medical University, Nanjing, China; 3 Department of Medical Oncology, Jiangsu Cancer Hospital, Jiangsu Institute of Cancer Research, The Affiliated Cancer Hospital of Nanjing Medical University, Nanjing, China

**Keywords:** circEIF3M, miR-33a, triple-negative breast cancer, CCND1

## Abstract

We investigated the function of circular RNA circEIF3M (hsa_circ_0003119) in triple-negative breast cancer. The expression profiles of circRNAs in 3 specimens of triple-negative breast cancer tissues with adjacent nontumor tissues were analyzed by RNA-sequencing. We verified the oncogenic role of circEIF3M in triple-negative breast cancer through a series of biological function experiments. It was found that circEIF3M was markedly upregulated in triple-negative breast cancer as compared to adjacent nontumor tissue, and that circEIF3M promoted triple-negative breast cancer cell proliferation, migration, and invasion. Mechanistic analysis indicated that circEIF3M may act as a competing endogenous RNA for miR-33a that relieves the inhibitory effect of miR-33a on its target cyclin D1. These findings showed that circEIF3M promotes triple-negative breast cancer progression via the circEIF3M/ miR-33a/ cyclin D1 axis.

## INTRODUCTION

Breast cancer is a progressive disease that is a leading cause of cancer-related mortality in women. Because the basal-like breast cancer subtype has the poorest prognosis [1], we focused on this subtype in this study. The basal-like subtype is generally negative for estrogen receptor (ER), progesterone receptor (PR) and human epidermal growth factor receptor-2 (HER2), and therefore also called triple-negative breast cancer (TNBC) [2]. Currently, targeted therapeutic strategies are available for breast cancers that are ER, PR or HER2 positive, but there are no specific targeted therapy options available for TNBC. Consequently, new treatments based on the specific mechanisms of TNBC are urgently needed to improve treatment efficacy and avoid the adverse effects of conventional therapies.

Recently, a novel class of noncoding RNAs, termed circular RNAs (circRNAs), has been attracting much attention. CircRNAs have been shown to regulate gene expression by acting as miRNA sponges [3]. For example, cerebellar degeneration associated protein 1 antisense transcript (CDR1as) contains more than 70 binding sites for miR-7 and acts as a miRNA sponge that strongly inhibits miR-7 activity within neuronal tissues, thereby increasing levels of miR-7 targets [3]. Recent research has also demonstrated the importance of circRNAs in regulating cancer-related signaling pathways [4, 5]. In addition, circRNAs may be related to specific tumor types and serve as predictive risk factors and prognostic factors for certain types of cancer [6, 7].

Little is currently known about the associations between circRNAs and TNBC. In the present study, therefore, we used high-throughput RNA sequencing (RNA-Seq) to screen the circRNA profiles of TNBC tumor tissues. This enabled us to detect a novel circRNA, circEIF3M, which promotes tumor cell progression by acting as a sponge for the miR-33a, thereby relieving the microRNA repression of target gene cyclin D1 (CCND1).

## RESULTS

### CircRNA and mRNA expression profiles in TNBC

**Figure 1:**
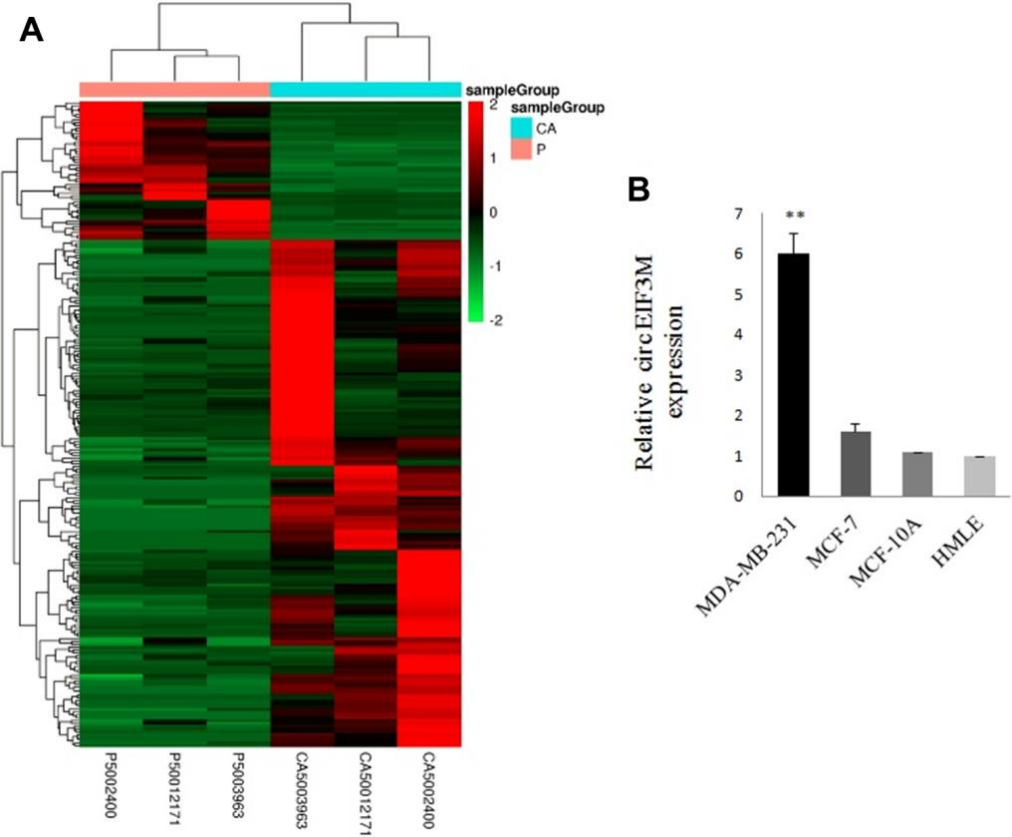
**Overview of circRNA profiles (CA: TNBC tissues; P: normal breast tissues).** (**A**) Hierarchical cluster analysis of differentially expressed circRNAs. The red and green colors indicate high and low expression, respectively. (**B**) Relative expression of circEIF3M in cell lines was determined by qRT-PCR. **P < 0.01.

A total of 34674 circRNAs were identified by RNA-Seq in three pairs of TNBC and normal breast tissue samples. The density of the length of these circRNAs is presented in Supplementary Figure 1. The raw read counts were normalized and differentially expressed circRNAs were filtered using DESeq2. We got 229 circRNAs with a p-value < 0.05, including 180 upregulated circRNAs and 49 downregulated circRNAs (Figure 1A and Supplementary Figure 2). All the 229 different expressed circRNAs are presented in Supplementary Table 1. The results revealed that expression of chr11:32589546:32596047:+ (circEIF3M) was the most upregulated. The qRT-PCR validation data performed in cell lines were strongly consistent with the circRNA sequencing data, indicating the reliability of the RNA-Seq results (Figure 1B). Moreover, 3668 mRNAs were differentially expressed and CCND1 was the most upregulated gene in TNBC tissues. Therefore, we focused on circEIF3M and CCND1.

### Prediction of circRNA/miRNA/ mRNA interaction

**Figure 2:**
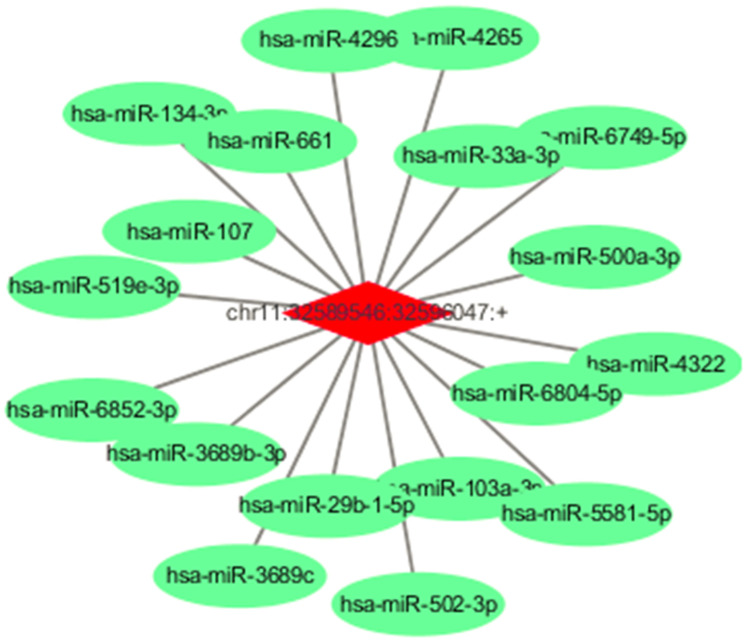
**Predicted miRNAs for the circEIF3M and predicted ceRNA for the dysregulated circRNAs.** Detailed annotation of circRNA-miRNA interactions between circEIF3M and its target miRNAs.

Because circRNAs contain corresponding miRNA binding sites and can act as miRNA sponges, to assess their potential functions in TNBC, we used miRNA target prediction software to investigate miRNAs intrinsically closely related to the identified differentially expressed circRNAs. The predicted miRNAs for the top one dysregulated circRNA are listed in Figure 2. For example, circRNA chr11:32589546:32596047:+ is predicted to harbor hsa-miR-33a-3p, hsa-miR-29b-1-5p, hsa-miR-107, and hsa-miR-134-3p. In addition, a circRNA-miRNA- mRNA network was constructed (Supplementary Figure 3), indicating a potential competing endogenous RNA (ceRNA) relationship among circRNA, miRNA, and mRNA in TNBC.

### CircEIF3M and CCND1 are upregulated in TNBC

**Figure 3:**
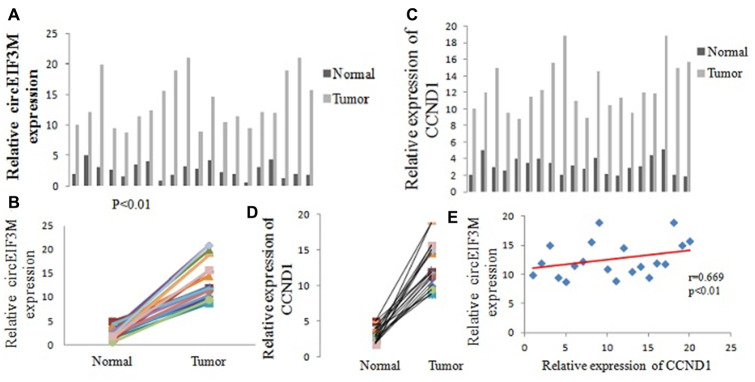
**CircEIF3M and CCND1 are upregulated in TNBC.** (**A** and **B**) Relative expression of circEIF3M in TNBC tissues (Tumor) and adjacent non-tumor tissues (Normal) was detected by qRT-PCR (n = 20). (**C** and **D**) Relative expression of CCND1 in TNBC tissues (Tumor) and adjacent non-tumor tissues (Normal) was detected by qRT-PCR (n =20). (**E**) Pearson correlation analysis of circEIF3M and CCND1 expression in 20 TNBC tissues. Data were showed as mean ± SD, P < 0.01.

The expressions of circEIF3M were detected by Quantitative real time polymerase chain reaction (qRT-PCR) in 20 pairs of TNBC tissues. According to our RNA-seq data, the results showed that circEIF3M was significantly upregulated in TNBC tissues compared with adjacent normal tissues (Figure 3A, 3B). The circRNA circEIF3M is an exonic circRNA and derived from the EIF3M gene Exon5, Exon6, Exon7 and Exon8 (Supplementary Figure 4). To confirm whether CCND1 is co-overexpressed with circEIF3M, the levels of CCND1 were detected in the 20 pairs of TNBC tissues by qRT-PCR. The results showed that CCND1 was also upregulated in TNBC (Figure 3C, 3D). Statistical analysis found that the expression of circEIF3M was positively correlated with that of the CCND1 (Figure 3E). These results confirmed our RNA-seq data and indicated that circEIF3M and CCND1 might be involved in the progression of TNBC.

### CircEIF3M promotes TNBC cell proliferation

**Figure 4:**
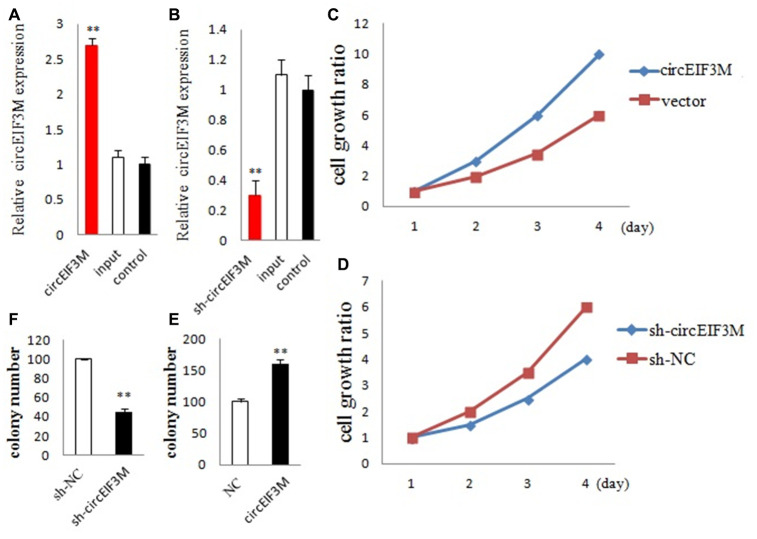
**CircEIF3M promotes TNBC cell proliferation.** (**A** and **B**) qRT-PCR analysis of circEIF3M expression in TNBC cells transfected with circEIF3M expression vector, sh-circ, input, or control. (**C** and **D**) The growth curves of cells transfected with indicated vectors were evaluated by CCK8 assays. (**E** and **F**) Colony formation assays were executed to detect the proliferation of cells transfected with indicated vectors. Data were showed as mean ± SD, **P < 0.01.

We detected the expression of circEIF3M and its parental gene EIF3M in the transfected cells. The circEIF3M was overexpressed and knocked down in MDA-MB-231 cells respectively. The expression of circEIF3M was significantly different from that of the control group (Figure 4A and 4B). However, there was no difference in the expression of EIF3M (Supplementary Figure 5), suggesting that the expression of circEIF3M did not activate or inhibit the expression level of EIF3M. To study the biological function of circEIF3M in TNBC cells, CCK8 assays were carried out to show that upregulation of circEIF3M significantly increased the proliferation capacity of MDA-MB-231 cells, while downregulation of circEIF3M inhibited cell growth (Figure 4C and 4D). Colony formation assays further confirmed that upregulation of circEIF3M can increase the proliferation of MDA-MB-231 cells, while downregulation of circEIF3M can significantly suppress the proliferation of MDA-MB-231 cells (Figure 4E and 4F). These results indicated that circEIF3M promotes proliferation of TNBC cells.

### CircEIF3M promotes TNBC cell progression

**Figure 5:**
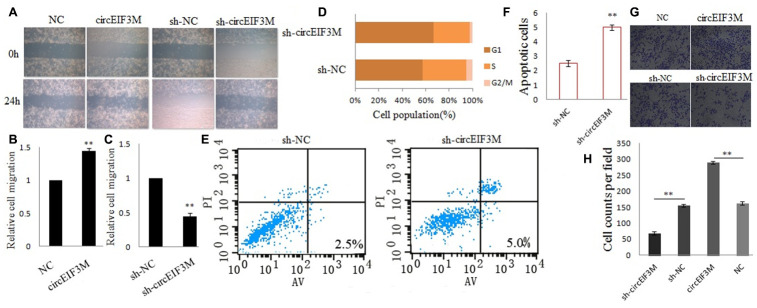
**CircEIF3M promotes TNBC cell progression.** (**A**–**C**) Cell migration capacities were detected by wound healing assays after transfected with indicated vectors (magnification, × 40, Scale bar, 200 μm.). (**D**) Cell cycle progression was analyzed by flow cytometry after transfected with indicated plasmids. (**E** and **F**) The apoptosis rate was analyzed by flow cytometry after downregulation of circEIF3M. (**G** and **H**) Cell invasion abilities were determined by transwell assays after transfection (magnification, × 100, scale bar, 100 μm). Data were showed as mean ± SD, **P < 0.01.

Transwell and wound healing assays were performed to explore the roles of circEIF3M in TNBC cells. The results suggested that up-regulation of circEIF3M significantly promoted the invasion and migration capacities of MDA-MB-231cells, whereas down-regulation of circEIF3M markedly inhibited the invasion and migration capacities of MDA-MB-231cells (Figure 5A–5C; 5G, 5H). In addition, to evaluate whether circEIF3M has an effect on cell cycle progression and apoptosis of TNBC cells, cell cycle analysis was carried out and the data revealed that downregulation of circEIF3M led to G1 arrest of TNBC cells (Figure 5D). Flow cytometry analysis further demonstrated that compared with the sh-NC control group, the apoptotic rate of MDA-MB-231 cells transfected with sh-circ was higher (Figure 5E, 5F). These data indicated that circEIF3M enhanced the progression of TNBC cells.

### CircEIF3M facilitates tumorigenesis of TNBC cells in vivo

**Figure 6:**
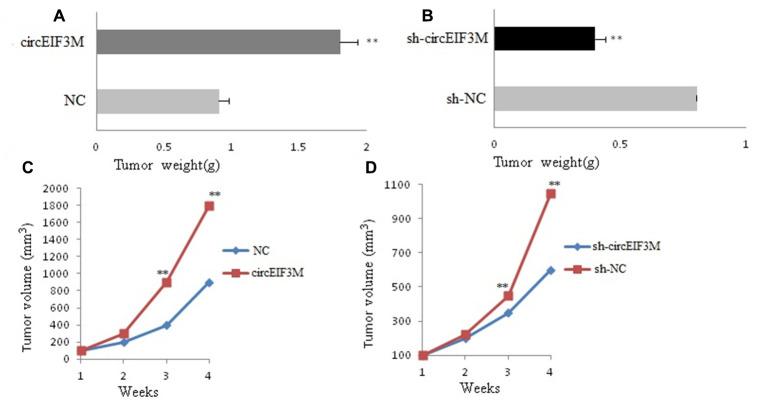
**CircAGFG1 facilitates tumorigenesis of TNBC cells in vivo.** (**A** and **B**) Tumor weight was shown. (**C** and **D**) Growth curves of xenograft tumors which were measured once a week. Data were indicated as mean ± SD, **P < 0.01.

**Figure 7:**
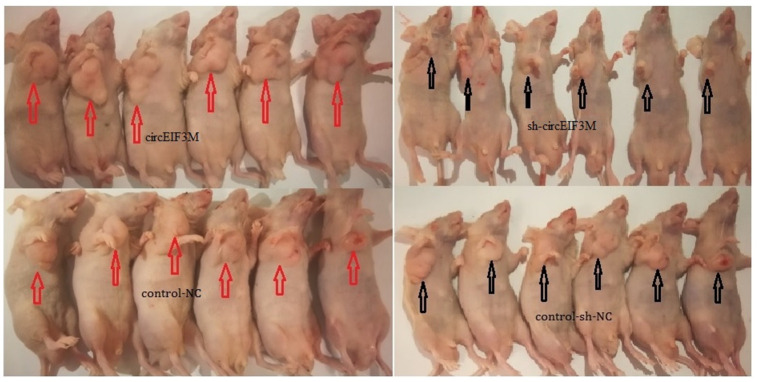
**Pictures of subcutaneous tumors were displayed.** The female nude mice were randomly divided into four groups: circEIF3M, sh-circEIF3M, control-NC and control-sh-NC (each group n=6).

In order to study the effects of circEIF3M on tumor growth in vivo, MDA-MB-231 cells were stably transfected with over expression or vector control and infected with sh-NC or sh-circ and then subcutaneously injected into nude mice. The results of animal experiments showed that compared with the control group, the tumors derived from cells overexpressing circEIF3M were bigger and heavier. Conversely, compared with the sh-NC control group, the tumors derived from cells infected with sh-circ had smaller sizes and lower weights (Figure 6A–6D; Figure 7). Taken together, the data confirmed the oncogenic role of circ EIF3M in TNBC.

### CircEIF3M acts as a sponge for miR-33a

To comprehend the molecular function of circEIF3M, we predicted the potential targets of circEIF3M by miRNA target prediction software (TargetScan database). We investigated the ability of chr11:32589546:32596047:+ binding miRNAs. MiRNA mimics of the 10 miRNAs that potentially interacted with chr11:32589546:32596047:+ were synthesized and cotransfected with psiCHECK2-circ, respectively. We mutated each miRNA target site from psiCHECK2-circ expressing vector, denoted psiCHECK2-circ-M. MiR-33a mimics cotransfected with psiCHECK2-circ had a reduced the luciferase reporter activities, compared with those cotransfected with psiCHECK2-circ-M (Supplementary Figure 6). The other 9 miRNA mimics and negative control of mimics showed no effect on luciferase reporter activities of neither psiCHECK2-circ nor psiCHECK2-circ-M.

**Figure 8:**
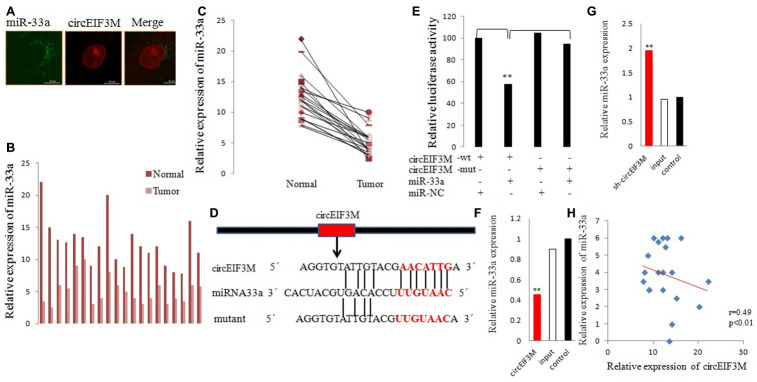
**CircEIF3M functions as a sponge for miR-33a.** (**A**) FISH was performed to observe the cellular location of circEIF3M (red) and miR-33a (green) in cells (magnification, × 200, scale bar, 100 μm). (**B** and **C**) Relative expression of miR-33a in TNBC tissues (Tumor) and adjacent non-tumor tissues (Normal) was determined by qRT-PCR (n = 20). (**D**) Schematic illustration of circEIF3M-WT and circEIF3M-Mut luciferase reporter vectors. (**E**) The relative luciferase activities were detected after transfection with circEIF3M-WT or circEIF3M-Mut and miR-33a mimics or miR-NC, respectively. (**F** and **G**) The relative expression of miR-33a was detected by qRT-PCR after transfection with indicated vectors. (**H**) Pearson correlation analysis of circEIF3M and miR-33a expression in 20 TNBC tissues. Data were indicated as mean ± SD, **P < 0.01.

We performed FISH assay to observe the localization of circEIF3M and miR-33a in TNBC cells, and found that most of circEIF3M (red) and miR-33a (green) were co-located in cytoplasm (Figure 8A). Then, the expression levels of miR-33a was detected in 20 pairs of TNBC tissues, and the results exhibited that miR-33a was significantly inhibited in TNBC tissues compared with normal tissues (Figure 8B, 8C). Furthermore, dual-luciferase reporter assay was conducted, and the results showed that miR-33a mimics could significantly suppress the luciferase activity of WT group but not the mutant one (Figure 8D, 8E), indicating that there was a direct correlation between circEIF3M and miR-33a. In addition, we found that overexpression of circEIF3M led to reduction in miR-33a, while down-regulation of circEIF3M may markedly increase miR-33a expression in MDA-MB-231 cells (Figure 8F, 8G). Statistical analysis showed a negative correlation between circEIF3M and miR-33a expression in 20 TNBC tumor tissue samples (Figure 8H). Together, these data proved that circEIF3M could function as a sponge for miR-33a.

### CircEIF3M/miR-33a/ CCND1 interaction

**Figure 9:**
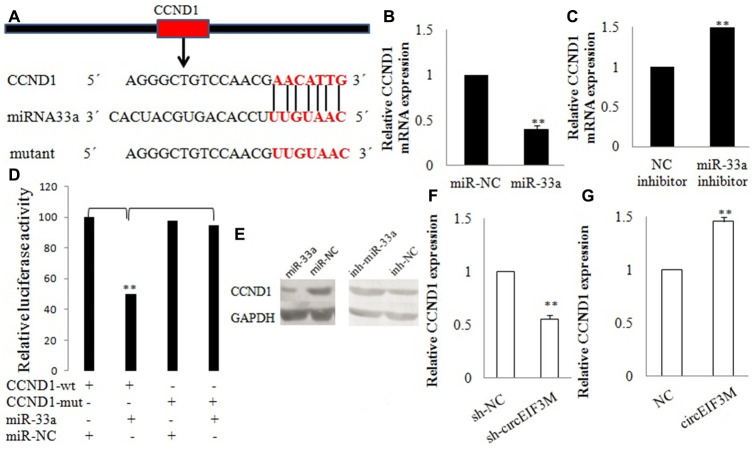
**CCND1 is directly targeted by miR-33a.** (**A**) Schematic illustration of CCND1 3’UTR-WT and CCND1 3’UTR-Mut luciferase reporter vectors. (**B**, **C** and **E**) Relative mRNA and protein levels of CCND1 were detected in cells after transfected with miR-NC, miR-33a, NC inhibitor and miR-33a inhibitor using qRT-PCR, respectively. (**D**) The relative luciferase activities were detected after transfected with CCND1 3’UTR-WT or CCND1 3’UTR-Mut and miR-33a mimics or miR-NC, respectively. (**F** and **G**) Relative expression of CCND1 was detected by qRT-PCR in cells transfected with indicated vectors, circEIF3M or sh-circEIF3M. Data were indicated as mean ± SD, **P < 0.01.

**Figure 10:**
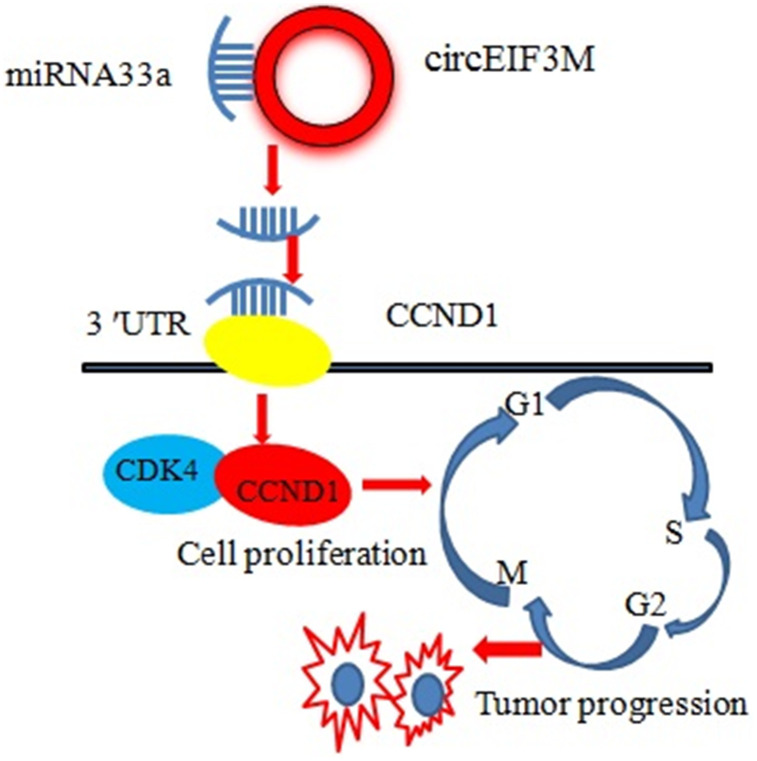
Schematic diagram of how circEIF3M promotes TNBC progression: Upregulation of circEIF3M is sponging miR-33 to increase CCND1.

CCND1 and circEIF3M share the same MRE of miR-33a according to target prediction software -TargetScan (Figure 9A). MiR-33a mimics significantly decreased the expression of CCND1, while miR-33a inhibitors significantly increased the expression of CCND1 in MDA-MB-231 cells (Figure 9B, 9C). A dual luciferase reporter assay was conducted to evaluate this prediction, and the data suggested that miR-33a mimics could suppress the activity of the luciferase reporter gene with CCND1 3’UTR-WT compared to the mutant group (Figure 9D). In addition, the protein expression level of CCND1 also changed correspondingly in TNBC cells (Figure 9E). As expected, knockdown of circEIF3M significantly inhibited the expressions of CCND1, while overexpression of circEIF3M greatly activated the expression level of CCND1 (Figure 9F–9G). In summary, these data show that circEIF3M could activate CCND1 expression through serving as a ceRNA for miR-33a (Figure 10).

## DISCUSSION

TNBC is a highly malignant breast cancer subtype with a poorer prognosis than other molecular subtypes. Despite the development therapeutic strategies that enable patients to achieve pathological complete response after neoadjuvant chemotherapy, TNBC patients still have higher metastasis rates and poorer prognoses than patients with other breast cancer subtypes. Therefore, in an effort to identify an effective therapeutic target for TNBC, we performed a comprehensive systemic bioinformatics analysis of the global changes in the pattern of circRNA expression in TNBC.

Only a few circRNAs have been functionally well characterized so far, and the biological functions of most circRNAs in breast cancer remain unknown. Growing evidence indicates that in multiple human cancers, circRNAs regulate the expression of miRNA target genes by acting as sponges for the miRNAs [3, 8, 9]. In the present study, we identified a novel circRNA, circEIF3M, which was highly upregulated in TNBC cells and tissues, and significantly promoted the progression of TNBC cells. We also showed that circEIF3M acts as a ceRNA for miR-33a to activate CCND1 expression, which led to progression of TNBC. These findings suggest that circEIF3M could potentially serve as a therapeutic target for TNBC.

Our bioinformatics analysis revealed that circEIF3M contains the MRE of miR-33a. Moreover, dual-luciferase reporter assays confirmed that circEIF3M directly interacts with miR-33a. We also found that miR-33a was significantly downregulated in TNBC tissues, and its levels correlated negatively with circEIF3M. Consistent with our results, miR-33a reportedly inhibits breast cancer invasion and metastasis [10]. Similarly, miR-33a may also serve as a tumor suppressor and is downregulated in other cancers, including liver, pancreatic and lung cancers [11–13]. Our findings thus suggest that circEIF3M acts as an oncogene by sponging miR-33a in TNBC.

In the light of ceRNA hypothesis, circRNAs act as ceRNAs to increase expression of miRNA target genes. We found that CCND1 and circEIF3M are both overexpressed in TNBC. Notably, our bioinformatics analysis showed that CCND1 is a potential target of miR-33a, while a dual-luciferase reporter assay proved that miR-33a can directly bind to the 3'-untranslated region (3′-UTR) of CCND1. In addition, upregulation of miR-33a led to downregulation of CCND1 at both the mRNA and protein levels, while downregulation of miR-33a had the opposite effect. It is well known that CCND1 mainly coordinates with cyclin-dependent kinase 4 (CDK4) to regulate cell cycle progression. It is well established that CCND1-CDK4 activity is dysregulated in multiple cancers, including melanoma and breast cancer [14, 15]. Coincidentally, it was found that downregulation of circEIF3M can lead to cell cycle arrest.

It has been confirmed that overexpression of CCND1 is associated with a poor prognostic factor in different type of cancers [14–18]. Consistent with earlier studies, our study also concluded that CCND1 was significantly upregulated in TNBC, and overexpression of CCND1 was related to poor prognosis. These findings support our hypothesis that circEIF3M acts as a ceRNA to promote CCND1-mediated proliferation and invasion by sponging miR-33a in TNBC. Moreover, they suggest that circEIF3M is a potential prognosis marker for TNBC, and that further analysis of circEIF3M/miR-33a/CCND1 signaling may contribute to a better understanding for the mechanism underlying TNBC progression.

## MATERIALS AND METHODS

### Patient samples

The 20 pairs of TNBC and adjacent normal breast tissues were recruited from patients who were diagnosed with TNBC at the Jiangsu Cancer Hospital, Jiangsu Institute of Cancer Research, Nanjing Medical University Affiliated Cancer Hospital. All the patients did not receive any anticancer treatment prior to surgery. All tissue samples were snap-frozen and stored in liquid nitrogen. All patients obtained written informed consent, and this study was approved by the ethics committee of the Jiangsu Cancer Hospital, Jiangsu Institute of Cancer Research, Nanjing Medical University Affiliated Cancer Hospital.

### RNA extraction and quality control

Total RNA was extracted with Trizol reagent according to the manufacturer’s instructions. A NanoDrop ND-2000 instrument (Thermo Fisher Scientific, USA) was used to evaluate the quality of the RNA samples and denaturing agarose gel electrophoresis was used to assess RNA integrity.

### qRT-PCR

Total RNA was isolated and 2 μg of total RNA was used in reverse transcription with PrimeScript RT Reagent Kit (Takara, Dalian, China) according to the manufacturer’s protocol. qRT-PCR was conducted on the ABI Prism 7500 system (Applied Biosystems, CA, USA) with SYBR Green Premix Ex Taq (Takara, Dalian, China). GAPDH was used as an internal control for circRNA and mRNA, while U6 for miRNA. The relative expression of genes was calculated using the 2-ΔΔCT method. Primers are presented in Supplementary Table 2.

### CircRNA sequencing and bioinformatic analysis

Three pairs of TNBC and adjacent normal breast tissues were selected for RNA-seq analysis. Total RNA from each sample was used to prepare circRNA sequencing libraries. Total RNA was extracted using the miRNeasy Mini Kit (Qiagen, Hilden, Germany). 1ug of total RNA from each sample was subjected to the NEBNext Ultra Directional RNA Library Prep Kit (NEB, USA) for Illumina to remove ribosomal RNA prior to the construction of the RNA-seq libraries, Strand-specific RNA-seq libraries were prepared using the NEBNext Ultra Directional RNA Library Prep Kit for Illumina. Briefly, RNA samples were fragmented and then used for first-strand and second-strand cDNA synthesis with random hexamer primers. For second-strand cDNA synthesis, dUTP mix (without dTTP) was used which allows for the removal of the second strand. The cDNA fragments were treated to repair the ends, then modified with Klenow to add an A at the 3′end of the DNA fragments, and finally ligated to adapters. Ligated cDNA products were purified and treated with Uracil-DNA Glycosylase to remove the second-strand cDNA. Then first-strand cDNA was subjected to 15 cycles of PCR amplification. Library quality was determined on Bioanalyzer 4200 (Agilent, Santa Clara, CA, USA). Then the strand-specific circRNA-seq libraries was sequenced in HiSeq X10 system (Illumina, San Diego, CA, USA) on a 150bp paired-end run. Briefly, DCC software was used to detect and annotate circRNAs [19], based on two public circRNA databases: circBase [20] and circ2Traits [21]. The R software was used to identify differentially expressed circRNAs [22]. CircRNAs exhibiting fold changes (FCs) ≥ 2.0 with P values < 0.05 were considered to be differentially expressed, and cytoscape software was used to construct the circRNA-miRNA network.

### Cell culture

Human TNBC cell lines (MDA-MB-231), MCF-7 and normal mammary epithelial cell line (MCF-10A, HMLE) were purchased from American Type Culture Collection (ATCC) (Manassas, VA, USA) and were authenticated by short tandem repeat profiling. These cell lines were maintained at 37 °C in 5% CO _2_ in DMEM (Gibco, Carlsbad, CA, USA), supplemented with 10% fetal bovine serum, 100 U/ml penicillin and 100 mg/ml streptomycin. All experiments were conducted with cells from passage numbers 5–15.

### Cell transfection

The construction of a lentiviral vector over-expressing circEIF3M, and a lentiviral vector expressing siRNA for circEIF3M were conducted by Geneseed Biotech (Guangzhou) Co., Ltd. Briefly, the full-length cDNA of circEIF3M was amplified and then cloned into over expression vector pLCDH-ciR. To knock down circEIF3M, a circEIF3M siRNA and a control siRNA-NC were synthesized. After efficiency examination by qRT-PCR, then the shRNA against circEIF3M and the control shRNA-NC were synthesized. The sequences of the siRNA and shRNA were listed in Supplementary Table 3. The miRNA mimics, inhibitors and negative controls were synthesized by GenePharma (Shanghai, China). Cell transfections were conducted with Lipofectamine 2000 (Invitrogen) according to the manufacturer’s protocols.

### Cell proliferation, cell cycle and apoptosis assays

Cell proliferation assays were performed by Cell Counting Kit-8 according to the manufacturer’s protocols. Cell cycle assays were performed by a flow cytometry (FACSCalibur, USA). The results were presented as the percentage of cells in each phase. Apoptosis assays were executed using double staining with fluorescein isothiocyanate (FITC)-conjugated Annexin V and propidium iodide (PI). Next, the percentages of cells were analyzed on a flow cytometer (FACSCalibur, USA). All experiments were repeated in triplicate.

### Transwell migration and invasion assays

Briefly, TNBC cells transiently overexpressing miR-33a or circEIF3M were incubated in transwell plates for 24 h. Then cells that migrated through an 8-μM pored membrane or invaded through the Matrigel-coated membrane were stained and counted under a microscope. Experiments were done in triplicate.

### Fluorescence in situ hybridization (FISH)

FISH was performed to detect the location of circEIF3M and miR-33a in TNBC cells. In short, cell slides were hybridized with specific Cy3-labeled circEIF3M probes and FITC-labeled miR-33a probes at 37 °C overnight, and then stained with DAPI. Finally, the images were obtained under a fluorescence microscope.

### Dual-luciferase reporter assay

The dual-luciferase reporter system was employed according to the manufacturer's instructions. Briefly, circEIF3M and CCND1–3’UTR and their mutant vectors were synthesized and subcloned into luciferase reporter vector psiCHECK2 (Promega). The relative luciferase activity was determined by the Dual Luciferase Assay Kit (Promega).

### Western blot analysis

The total proteins of cells were extracted and subjected to western blot analysis as described previously [23]. Antibodies were as follows: mouse anti-CCND1, mouse anti-GAPDH (Cell Signaling Technology, USA) and anti-mouse secondary antibodies (Santa Cruz Biotechnology). Molecular sizes of CCND1 and GAPDH proteins shown on the immunoblots are 36kD and 146kD, respectively. Each experiment was done in triplicate.

### In vivo experiments

All animal experiments were approved by Nanjing Medical University Institutional Animal Care and Use Committee. Twenty-four female nude mice were randomly divided into four groups: circEIF3M, sh-circEIF3M, control-NC and control-sh-NC (n=6 each group). Briefly, for xenograft experiments, 1 × 10^7^ MDA-MB-231 cells were injected subcutaneously into the mammary fat pads of nude mice. There were six mice per group. Tumor size was monitored once per week. The formula for calculating the volume of tumors was 0.5×length×width^2^. The mice were sacrificed after 4 weeks and tumors tissues were removed for further analysis.

### Statistical analysis

Statistical analyses were performed by SPSS 17.0. Data were showed as mean ± standard deviation. The differences between groups were assessed using paired or unpaired t test (two-tailed). The correlation between groups was analyzed by Pearson correlation. **P value < 0.01 was considered as statistically significant.

## Supplementary Material

Supplementary Figures

Supplementary Table 1

Supplementary Tables 2, 3
