# Supplementary material for: The circular RNA circEIF3M promotes breast cancer progression by promoting cyclin D1 expression
Source: Aging (Albany NY). 2020 Jul 11;12(14):14775–90. doi: 10.18632/aging.103539 (PMC7425499; doi:10.18632/aging.103539)
Supplement: Supplementary Figures [file aging-12-103539-s003..pdf]

SUPPLEMENTARY FIGURES

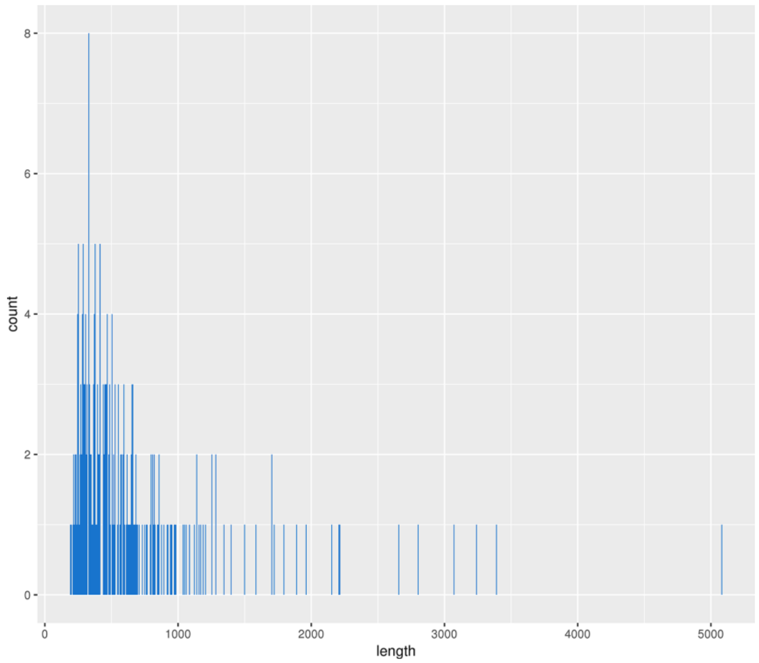

Supplementary Figure 1. Density of spliced length of the detected circRNAs.

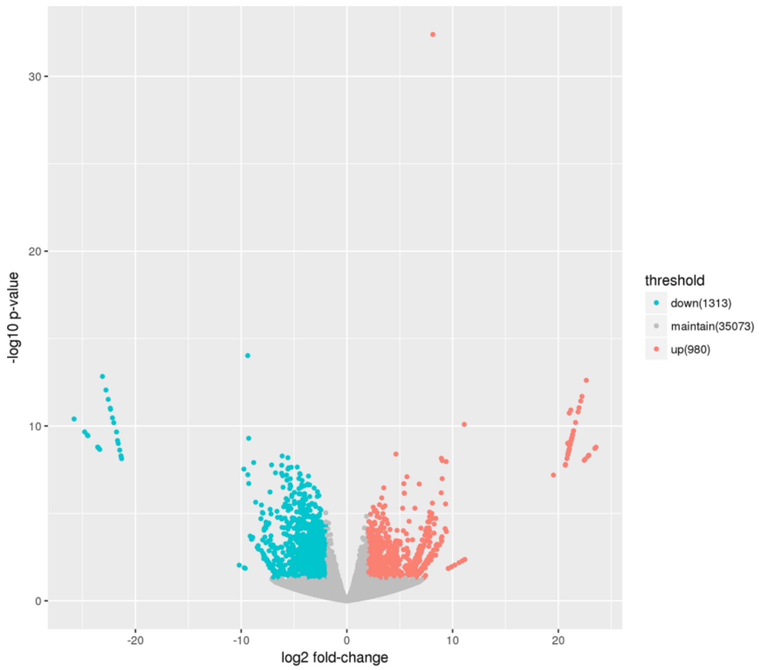

Supplementary Figure 2. Volcano plot of differentially expressed circRNAs in TNBC. The vertical green lines correspond to two-fold increased and decreased expression, and the horizontal green line represents. The red points represent circRNAs that were differentially expressed with statistical significance.

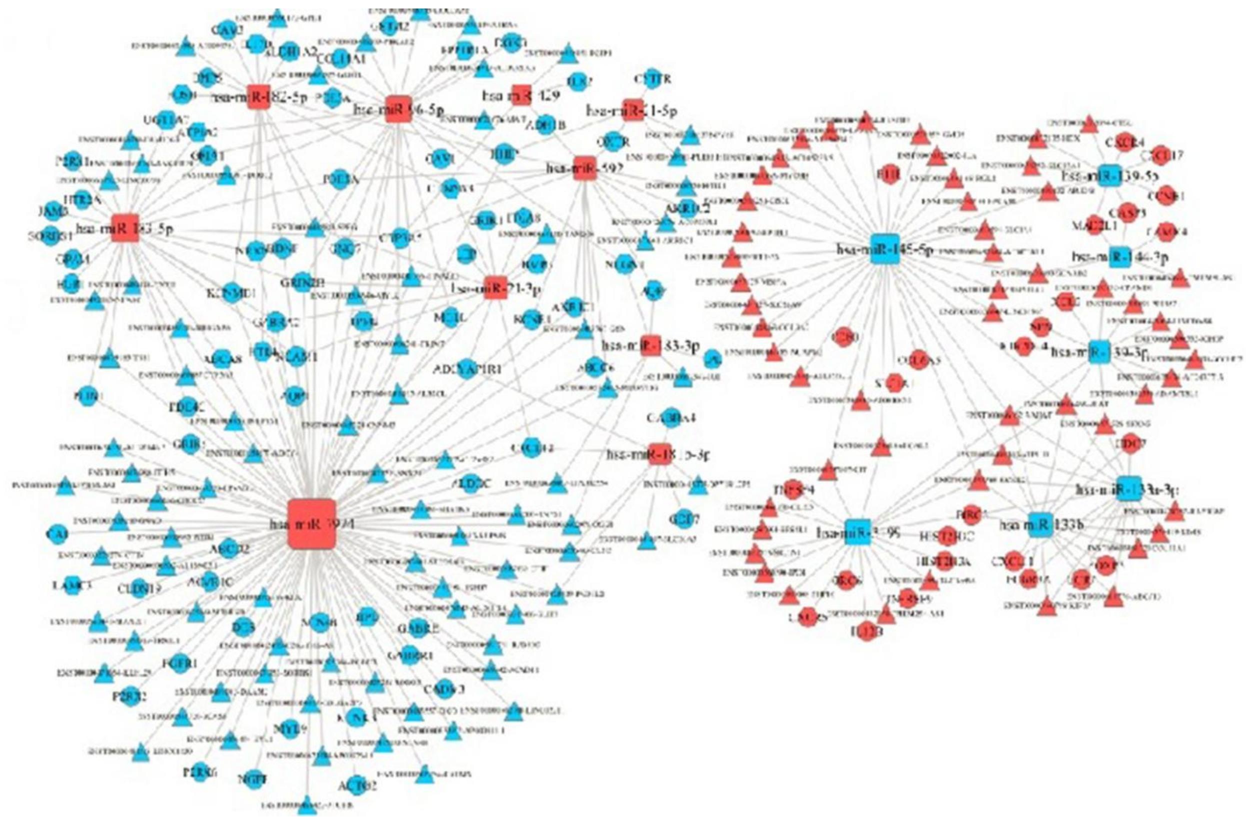

Supplementary Figure 3. Detailed annotation of predicted circRNA-miRNA-mRNA interaction network for the dysregulated circRNAs.

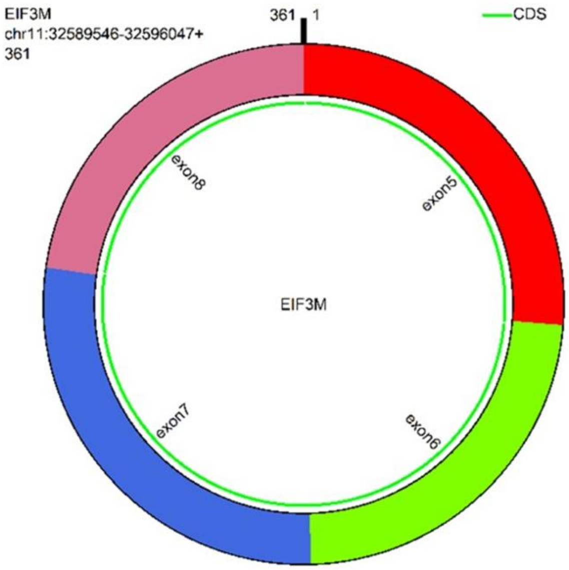

Supplementary Figure 4. The annotation result for chr11:32589546:32596047:+. Through gene sequence alignment, we found that this CircRNA ID of chr11:32589546:32596047:+ is hsa_circ_0003119.

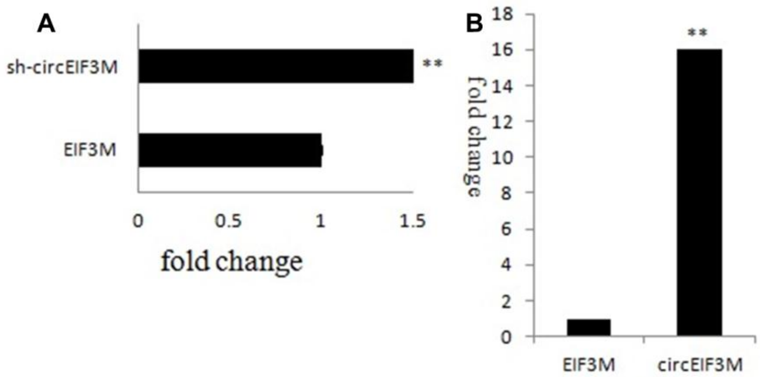

Supplementary Figure 5. The fold changes of circEIF3M and EIF3M gene in 231 cells after transfection against control cells. The circEIF3M was knocked down (**A**) and overexpressed (**B**) in MDA-MB-231 cells respectively. The relative expression of circEIF3M and EIF3M were detected by qRT-PCR after transfection with indicated vectors.

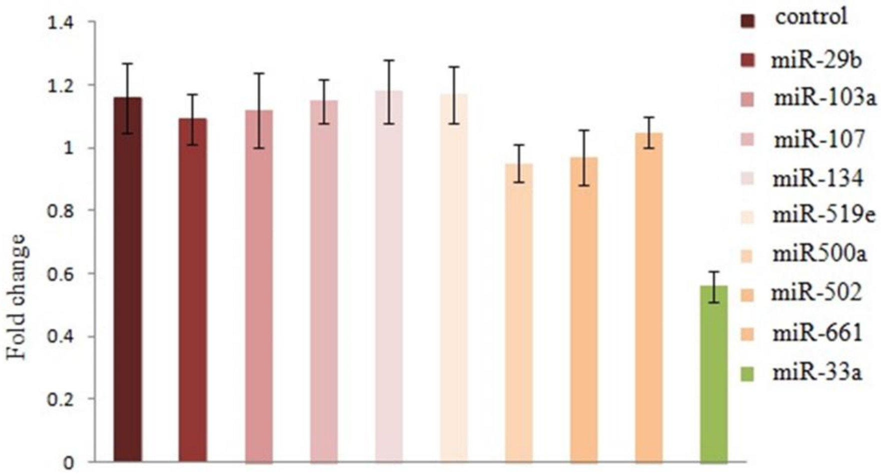

Supplementary Figure 6. Luciferase reporter assay for the luciferase activity of psiCHECK2-circ against psiCHECK2-circ-M co-transfected with miRNA mimics or negative control of mimics.
