# Supplementary material for: The circular RNA circEIF3M promotes breast cancer progression by promoting cyclin D1 expression
Source: Aging (Albany NY). 2020 Jul 11;12(14):14775–90. doi: 10.18632/aging.103539 (PMC7425499; doi:10.18632/aging.103539)
Supplement: Supplementary Tables 2, 3 [file aging-12-103539-s002..pdf]

SUPPLEMENTARY TABLES

Supplementary Table 2. Primers for qRT-PCR analysis.

Name	Sequences (5'—3')
qRT-PCR primers*	
miR-33a	F: GGGGGTGCATTGTAGTTG; R: TGCGTGTCGTGGAGTC
U6	F: CGAGCACAGAATCGCTTCA; R: CTCGCTTCGGCAGCACATAT
CCND1	F: CCCTCGGTGCCTACTTCAA; R: CTCCTCGCACTTCTGTTCCT
Hsa_circ_0003119	Divergent primers:<br>F: CTCTTCCTCTTGACCCTGCA; R: GTCCATACTCATCCTCCTGGG
GAPDH	Divergent primers:<br>F: TCCCCCACCACACTGAATCT; R: AACAGGAGGAGCAGAGAGCG

* F, forward; R, reverse.

Supplementary Table 3. Sequences of siRNAs and shRNAs used in this study.

Definition	sequences
siRNA	TCATTGATTCACTTGGTTAGA
si-NC	TTCTCCGAACGTGTCACGT
sh-circ	5’-GTTAACTCATTGATTCACTTGGTTAGATCAAGAGTCTAACCAAGTGA<br>ATCAATGATTTTTTCTCGAG-3’
sh-NC	5’-TTCTCCGAACGTGTCACGTTCAAGAGACGTGACACGTTCGGAGAATTTTTT-3’
